# WO_3_ Nanowires Enhance Molecular Alignment and Optical Anisotropy in Electrospun Nanocomposite Fibers: Implications for Hybrid Light-Emitting Systems

**DOI:** 10.1021/acsanm.1c04110

**Published:** 2022-03-09

**Authors:** Israel Greenfeld, Andrea Camposeo, Alberto Portone, Luigi Romano, Maria Allegrini, Francesco Fuso, Dario Pisignano, H. Daniel Wagner

**Affiliations:** † Department of Molecular Chemistry and Materials Science, Weizmann Institute of Science, Rehovot 76100, Israel; ‡ NEST, Istituto Nanoscienze-CNR and Scuola Normale Superiore, Piazza San Silvestro 12, Pisa I-56127, Italy; § Dipartimento di Fisica, Università di Pisa, Largo B. Pontecorvo 3, Pisa I-56127, Italy

**Keywords:** conjugated polymer, nanowire, NW, electrospinning, molecular orientation, adsorption

## Abstract

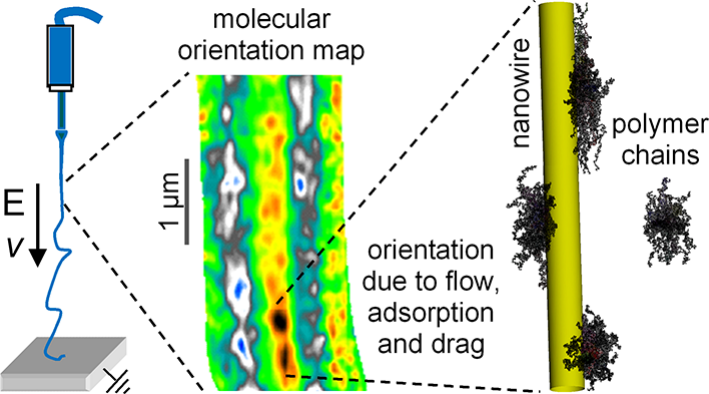

The molecular orientation in polymer fibers is investigated for the purpose of enhancing their optical properties through nanoscale control by nanowires mixed in electrospun solutions. A prototypical system, consisting of a conjugated polymer blended with polyvinylpyrrolidone, mixed with WO_3_ nanowires, is analyzed. A critical strain rate of the electrospinning jet is determined by theoretical modeling at which point the polymer network undergoes a stretch transition in the fiber direction, resulting in a high molecular orientation that is partially retained after solidification. Nearing a nanowire boundary, local adsorption of the polymer and hydrodynamic drag further enhance the molecular orientation. These theoretical predictions are supported by polarized scanning near-field optical microscopy experiments, where the dichroic ratio of the light transmitted by the fiber provides evidence of increased orientation nearby nanowires. The addition of nanowires to enhance molecular alignment in polymer fibers might consequently enhance properties such as photoluminescence quantum yield, polarized emission, and tailored energy migration, exploitable in light-emitting photonic and optoelectronic devices and for sensing applications.

## 1 Introduction

Polymer nanofibers are valuable building blocks for a variety of applications, ranging from smart textiles to electronics and photonics.^1−3^ They can be arranged in complex networks, whose topology can be exploited to yield an additional degree of freedom and thereby enhance specific properties, as recently reported for nanophotonic network lasers^4^ and for cell differentiation.^5^ The nanoscale arrangement of polymer macromolecules in fibers is also highly important for applications. For instance, nanofibers undergo substantial elongation during the electrospinning process, resulting in diameter shrinkage and preferred molecular orientation in the direction of the extensional flow.^6,7^ Rapid solvent evaporation, accelerated by the high surface-to-volume ratio in such jets, leads to rapid nanofiber solidification that retains the oriented conformation. In the resulting fibers, the mechanical, electrical, and optical characteristics of polymers may so be enhanced compared to the bulk.^8−10^ Importantly, further effects can be obtained by mixing hard fillers, such as nanowires (NWs), nanotubes, or nanoparticles, with the polymer solution.^11−17^ The question arises whether such fillers influence the polymer molecular orientation and properties in the resulting solid nanofibers, and if so, what are the physical mechanisms involved?

The effects of such local variations in molecular conformation in electrospun conjugated polymers are especially interesting. Given the conjugated bonding along their chain backbone,^8,9^ these polymers are useful for light-emitting diodes,^18^ lasers,^19^ and field effect transistors.^20^ The molecular conformation of conjugated polymers, specifically the length, orientation, and density of their conjugated chain segments, has a strong impact on their photophysical properties.^21−26^ For example, when the polymer chromophores (molecular parts that generate color) are aligned along the axis of a nanofiber, the photoluminescence quantum yield and the degree of polarization of the emission are significantly enhanced, leading to miniaturized light sources with improved signal-to-noise ratios of detected optical signals.^27^ Moreover, hybrid systems composed of organic semiconductors and of inorganic nanowires with complementary properties have been exploited in solar cells, light-emitting diodes, electrochromic devices, and photocatalytic systems.^28,29^ Whenever adding NWs to electrospun solutions leads to enhanced alignment of conjugated polymers, this can potentially improve the ability to funnel light to chromophores oriented along the fiber length,^30^ a condition highly desirable for light-harvesting applications,^31^ and for enhancing the polarization degree of the light emitted by the hybrid nanofibers.

The interface between solid fillers and a solution entails adsorption and hydrodynamic interactions, which affect the polymer molecular conformation. Generally, in the vicinity of an obstacle such as a filler, polymer chains in a solution are confined by the obstacle boundary in the directions normal to it. In terms of chain conformation, this means that the probability that the chain will expand in that direction is low. Consequently, the probability of chain expansion in the other directions is augmented. In the case of NW fillers, both the chain extension and orientation might increase in the direction along the NW. In addition, close to a filler boundary, the flow of the electrospun jet may be affected. The flow gradient (strain rate) that is responsible for chain extension can be locally decreased or increased by the hydrodynamic friction (drag) exerted by obstacles, thereby impacting molecular orientation.

The interaction between polymer chains and a confining surface and its effect on chain conformation have been a long standing topic in polymer physics since the seminal works of Flory and de Gennes on polymer chain adsorption, using scaling and mean-field calculations.^32,33^ These calculations showed that chains adsorbed by an attracting surface crowd near the surface into a flat pancake shape, to compensate for the loss of entropy due to confinement. Recent studies on polymer interactions with nanoparticles, by small-angle neutron scattering of polystyrene solutions loaded with silica nanoparticles,^34,35^ and by molecular dynamics simulation of polymer solution with nanorods,^36^ demonstrated extended conformation of chains close to a filler boundary. Electrospinning of poly(ε-caprolactone) solutions with multiwalled CNTs exhibited the nucleation and formation of chain-folded crystals on the CNTs, with distinct orientation.^37^ However, these studies do not provide simple and practical rules for achieving desired or optimal molecular orientation for optical applications by electrospinning. Therefore, it is our intention to offer a theoretical analytical solution, based on a scaling approach, which correlates the extensional flow conditions of electrospinning with the molecular orientation induced by chain extension, and validate it by optical measurements.

Here, we develop a theoretical model and study the exemplary solution of poly[2-methoxy-5-(2-ethylhexyloxy)-1,4-phenylene-vinylene] (MEH-PPV) and polyvinylpyrrolidone (PVP) added with tungsten trioxide (WO_3_) NWs. Blending conjugated polymers, which typically have rigid backbones and relatively low molecular weight and level of entanglement, with PVP, turns out to help obtain electrospun fibers without defects or beads.^38^ Moreover, in light-emitting conjugated polymers, the intrachain and interchain coupling between chromophores can favor the energy transfer from the photoexcited chromophores toward those with a lower energy, resulting in red-shifted emission. Such funneling of the excitation toward the chromophores with a lower energy is also expected to decrease the polarization degree of the emission.^39^ Therefore, the presence of PVP may also be beneficial for improving the polarization degree of light emission because of a decrease of interchain coupling in MEH-PPV.

Chain extension is analyzed by calculating the stretching and retracting dynamics of chains and the different effect on each polymer species in the blend. Monomer alignment probabilities are used to assess the adsorbing effect of obstacle boundaries, and the hydrodynamic drag induced by the NWs is used to assess the local increase or decrease of the strain rate. Effects are combined to calculate and map the molecular orientation at variable distances from NWs. Experimental evidence is obtained from nanofibers electrospun from the PVP/MEH-PPV/WO_3_ mixture and collected on a rotating drum. The spatial variation of molecular anisotropy is analyzed by polarized scanning near-field optical microscopy (SNOM), which provides maps of the optical dichroic ratio of MEH-PPV, supporting the theoretical predictions.

The results demonstrate that the incorporation of nanowires in nanofibers made of conjugated polymers turns out to be effective for enhancing the alignment of the polymer chains at the nanoscale, compared to neat polymer nanofibers. As a result, these nanowire-in-nanofiber hybrids are highly suitable for investigating nonradiative energy transfer and charge separation effects in nanocomposite systems and for exploitation as building blocks of miniaturized light-emitting devices with polarized emission and light-harvesting devices. The comprehensive rationalization of the molecular alignment mechanisms in the nanocomposite nanofibers demonstrated here can establish new design rules for next light-emitting nanostructures realized by electrospinning technologies.

## 2 Experimental Methods

### 2.1 Electrospinning

Solutions are prepared by dissolving 30 mg of MEH-PPV (*M*_W_ = 150–250 kDa, Sigma Aldrich) and 30 mg of PVP (*M*_W_ = 1.3 × 10^6^ Da, Alfa Aesar) in 1 mL of a mixture of chloroform (CHCl_3_, Sigma Aldrich) and dimethyl sulfoxide (DMSO, Sigma Aldrich) with a volumetric ratio of 9:1 (v/v). The solutions are stirred for 24 h. Next, WO_3_ NWs (Sigma Aldrich) are added to the solution to obtain a weight ratio of 20% with respect to the dry weight of the PVP/MEH-PPV mixture. The solutions are sonicated for 2 h and then injected through a 21G needle with a flow rate of 2 mL/h by using a programmable syringe pump (Harvard Apparatus). A 7 kV voltage bias (EL60R0.6-22, Glassman High Voltage) is applied between the needle and a drum with a grounded disk (8 cm diameter and 1 cm thickness) rotating at 2000 rpm and positioned at a distance of 5 cm from the needle. For optical characterization, fibers are deposited on quartz substrates (1 × 1 cm^2^, thickness 1 mm).

### 2.2 Morphological and Optical Characterization

The morphology of the composite fibers is investigated using an FEG-SEM (Merlin, Zeiss) at acceleration voltages in the interval 1.5–10 kV. Fluorescence confocal microscopy is carried out by using laser scanning (Olympus FV1000) coupled to an inverted microscope. Samples are excited using an Ar^+^ laser (excitation wavelength, λ_exc_ = 488 nm) through a 20× objective (numerical aperture, NA = 0.75). The fluorescence is collected by the same objective in a backscattering configuration, and the intensity is measured with a photomultiplier. In addition, the intensity of the excitation light transmitted by the sample is measured in each scan with a second photomultiplier.

### 2.3 Polarized Near-Field Microscopy

The nanoscale optical anisotropy of the nanocomposite fibers is investigated by polarization-modulation SNOM. Details of this technique are reported in our previous study.^9^ The microscope operates in the emission mode, where a probe made of a tapered optical fiber (LovaLite E50, nominal diameter apical aperture 50 nm) illuminates the sample in the near field, while the signal transmitted by the sample is collected using an aspheric lens and measured with a miniaturized photomultiplier (Hamamatsu R-9880). Topography maps, *h*(*x*, *y*), representing the local thickness of the sample, are measured simultaneously with the optical signal in each scan by the shear-force method. For nanoscale optical imaging, a solid-state laser (Roithner Lasertechnik MBL, λ = 473 nm), modulated in amplitude and polarization, is coupled to the tapered fiber. The polarization of the laser is controlled by means of a photoelastic modulator (PEM-100, Hinds Instruments), which operates as a waveplate with a retardation modulated sinusoidally at a frequency, *f* = 50 kHz. As a result, the ratio between polarization components of the light coupled into the probe optical fiber, measured along two mutually orthogonal directions, acquires a 2*f* modulation. The amplitude of the laser is instead modulated at frequency f* (f*/*f* < 0.1) by using a chopper (Scitec Instruments 300 CD). The photomultiplier is connected to two different digital dual lock-in amplifiers (Stanford Research SR830DSP): while the first one, which is referenced to f*, is used to obtain the optical transmission signal averaged over all polarization states (*I*_DC_), the second lock-in that is referenced to 2*f* provides an output signal (*I*_AC_) representative of the sample response to polarized radiation. The extinction coefficient is then evaluated through the expression *T*_NF_(*x*, *y*) = – ln [*I*_DC_(*x*, *y*)]/*h*(*x*, *y*), whereas the dichroic ratio of the sample, , where *I*_∥_ and *I*_⊥_ are the transmitted intensities for polarization aligned along two mutually orthogonal directions, is quantitatively evaluated from the ratio *I*_AC_/*I*_DC_. While this ratio accounts for local variations of the intensity of nonpolarized scattered light due, for instance, to near-field interaction with the NWs, contributions given by polarization-dependent light scattering by the NWs cannot be completely ruled out. As discussed previously,^9^ polarization modulation measurements require us to model the behavior of the entire optical system and to account for the residual optical activity of its components, including the optical fiber probe, eventually leading to an offset in γ. Since this offset remains constant during the SNOM scan, it can be determined and subsequently subtracted by performing reference measurements on bare substrates. The correct operation of the entire system is assessed through measurements of polarizing materials oriented along different directions.

## 3 Theoretical Modeling

### 3.1 Chain Extension and Adsorption during Electrospinning

**Figure 1:**
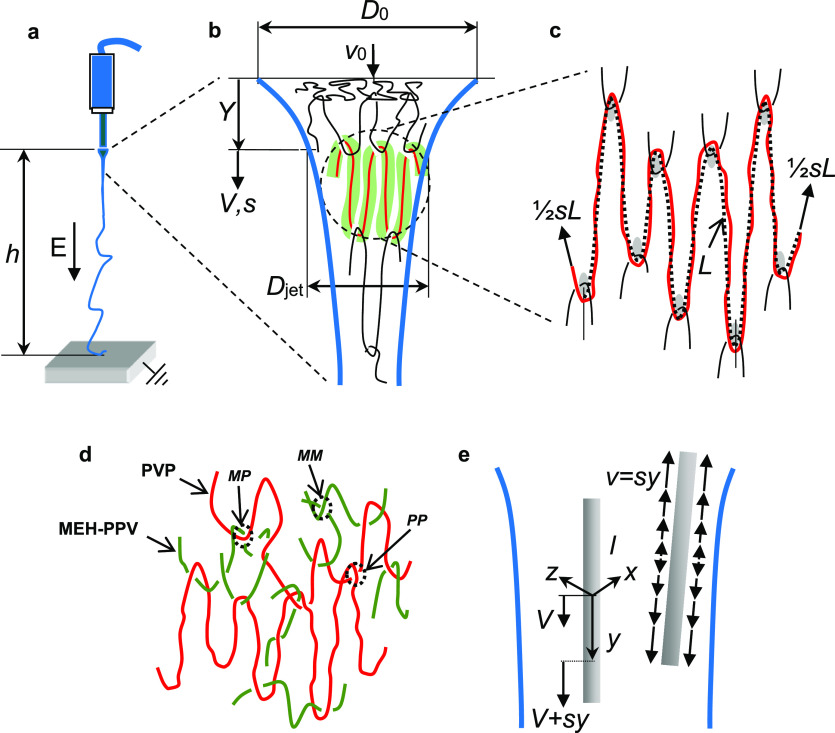
Polymer chain and filler dynamics in extensional flow. (a) Electrospinning at electrostatic field *E* across a gap *h*. (b) Stretched chain (red) and chain confining tube (green). *V* and *s* are the jet velocity and its constant gradient, respectively. (c**)** Chain (red) and its primitive path *L* (dotted line); the thick arrows indicate the elongation rate of the chain. (d) Network illustration of a blend of MEH-PPV (green) and PVP (red) polymers; three types of entanglement are marked: PP (PVP/PVP), MM (MEH-PPV/MEH-PPV), and MP (MEH-PPV/PVP). (e) NW and solution velocity *V* at *y* = 0, and solution velocity *V* + *ys* at the local (NW) position *y*; on the right, solution velocity *v* = *sy* relative to the NW (local velocity). The lateral *x* and *z* axes are also shown.

The polymer chains in an electrospun semidilute solution form an entangled network, consisting of strands extending between entanglement nodes (Figure 1a–c). Entanglements, which are topological constraints that prevent intercrossing of chains, are essential for spinnability of a polymer solution, ensuring continuity of the polymer network and the jet. When stretched under the stresses induced by the extensional flow, the entangled polymer network elongates elastically. In turn, the elastic forces in chains partially retract them, relieving some of the stress and achieving a new state of equilibrium.^40−44^ During extension and retraction, the mobility of a chain is constrained by the potential induced by nearby chains and is thus confined within a tubelike volume, a confining tube (Figure 1b), described by the known tube model.^36^ A chain reptates along the tube in a diffusive way, through thermal fluctuations of its monomers within the tube’s constraining diameter.^35^

The concepts of confining tubes, chain reptation, and chain stretching and retraction have been widely used in modeling the rheology of semidilute entangled polymer solutions under extensional flow.^33,45−51^ For a fast uniaxial flow such as electrospinning, the competing dynamics of chain extension and retraction described by these models can be simplified as the tube tends to align with the direction of the flow. Consequently, both the chain and its tube extend together affinely with the network, and when the chain retracts, its tube contracts along with it, so that the chain remains in the tube throughout extension and retraction.^45^ Consider a uniaxial flow with a strong time-dependent velocity gradient ∇*v* (that is, the strain rate). The polymer network is stretched affinely with the flow, resulting in relative extension of chains defined by ε = *L*/*L*_max_, where *L* is the primitive path of a chain (tube contour length, dotted line in Figure 1c) and *L*_max_ is the length of a fully extended chain (chain contour length). Thus, the chain extends at a rate of ε∇*v* due to affine convection. The ensuing net tension force in a stretched chain is *f*(ε) – *f*(ε_0_), where *f*(ε) is the normalized (dimensionless) elastic tension at an extension ε, and *f*(ε_0_) is the tension at equilibrium (at rest). This force tends to retract the chain through the tube back to equilibrium, within the Rouse relaxation time τ. Thus, the chain retracts at a rate of −[*f*(ε) – *f*(ε_0_)]/τ due to relaxation. Combining the stretching and retraction rates, the rate of the relative extension is given by (details in Supporting Information Section S1):^10^
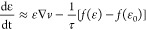
1The ≈ symbol denotes a scaling relationship, where constants of order unity are omitted.

The velocity gradient of the jet stream is fairly constant in a process,^10,52^ typically within the range of *s*∼10^3^–10^5^ s^–1^. When the electrospinning jet reaches the steady state, the constant velocity gradient is ∇*v* = *s*. Furthermore, since stretching and retraction reach equilibrium, the time derivative of the relative extension is zero. Consequently, eq 1 reduces to
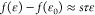
2This equation captures an essential property of the viscoelastic solution jet, namely, that the stress is proportional to the strain rate and the relaxation time. When both are high, the stress is high, and vice versa. When the relaxation time is short, for example, in short chains (small *N*), the strain rate must be very high to reach substantial stress, and vice versa. In other words, the stress is the result of competition between two time scales, that of the jet strain (s^–1^) and that of the chain relaxation (τ). The term *s*τ represents the instantaneous stiffness of the polymer liquid.

The network extension induced by the flow generates elastic tension in the chains. The elasticity of a polymer chain is entropic, and therefore, its tension is linear at small extension but rises sharply at high extension associated with less probable chain conformations. Thus, the chain extension dependence of the tension force is nonlinear, and several approximations are known.^32,48,50,53^ We use the following approximation for the elastic retraction force^10^
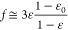
3which reflects the nonlinear behavior (adapted from Ianniruberto and Marrucci,^48^ see Supporting Information Section S1). Substituting the elastic force expression into eq 2 and solving for the relative chain extension, we obtain
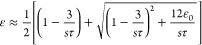
4Scaling approximations at small ε_0_ yield ε ≈ ε_0_(1 + *s*τ/3) for low *s*τ and ε ≈ 1 – 3/*s*τ for high *s*τ (see Supporting Information Section S4). The calculation of the relaxation time τ and initial extension ε_0_ for given polymer and solution properties is provided in Supporting Information Section S2.

Near the boundary of a filler such as an NW, a chain tends to adsorb by increasing the number of contacts with the surface in order to compensate for the loss of entropy due to confinement, consequently assuming the form of a flat pancake.^32,33^ The crowding of chains near a surface gradually decays at a characteristic distance from the surface, in the order of the polymer network correlation length. In other words, chains near the boundary are confined by the surface in one of the lateral directions due to adsorption and therefore essentially tend toward a two-dimensional conformation. The corresponding physical properties in the 2D condition are marked by an overbar accent. The expression for the elastic tension force changes to *f̅* ≅ 2ε̅(1 – ε̅_0_)/(1 – ε̅), where the prefactor 3 (eq 3) is replaced by 2 because the dimensionality is reduced from 3 to 2. The corresponding chain extension ε̅ is given by
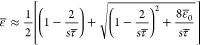
5

Scaling approximations at small ε̅_0_ yield ε̅ ≈ ε̅_0_(1 + *s*τ̅/2 ) for low *s*τ and ε̅ ≈ 1 – 2/*s*τ̅ for high *s*τ (see Supporting Information Section S4). These extended chain conformations are partially frozen upon nanofiber solidification because of the rapid solvent evaporation characteristic of electrospinning.

**Figure 2:**
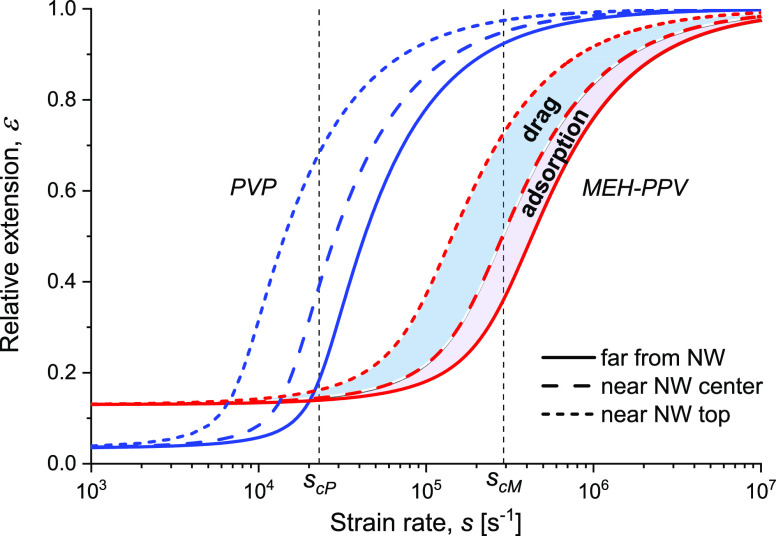
Chain relative extension in an electrospun polymer blend. Log-linear plot of the extension ε [eqs 4, 5 and 12] for each polymer species (PVP and MEH-PPV), depicted as a function of the electrospinning strain rate *s*, for the polymer blend specified in Table 1. The critical strain rates *s*_cP_ (PVP) and *s*_cM_ (MEH-PPV) are indicated. The unconfined 3D extensions are depicted by solid lines; the region marked as “adsorption” denotes extensions close to the NW center; the region marked as “drag” denotes extensions close to the NW top, where both adsorption and flow drag are significant.

When a polymer chain is dissolved in a solution and is free of external loads, it assumes a compact coil shape. The extension expressions in eqs 4 and 5 exhibit a transition, characterized by a steep extensional increase from a nearly coiled shape to a substantially extended shape (Figure 2). The transition occurs at a critical strain rate, defined by
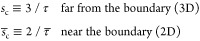
6

The critical strain rate determines such a transition: at low strain rates, the equilibrium between chain stretching and retraction is satisfied by small extensions; however, at high strain rates, the retraction stress must be high to balance stretching, leading to high extensions. High chain extension induces a high molecular alignment, which in turn enhances the material properties in the stretching direction.^10^ By using eq 6, eqs 4, 5 can be expressed in terms of the relative strain rates *s*/*s*_c_ = *s*τ/3 and *s*/*s̅*_c_ = *s*τ̅/2, respectively.

Throughout our calculations, we use the approximation τ̅ ≅ τ, based on the assertion that the friction experienced by a retracting chain is similar in both 2D and 3D conditions. To be exact, near a filler boundary, the monomer concentration slightly increases due to adsorption,^32,33^ resulting in a local increase in relaxation time (see the concentration dependence of τ in Supporting Information Section S2). The effect would be a left shift of the adsorption limit in Figure 2 (dashed curves), depending on the degree of adsorption related to the characteristic adsorption energy per monomer of each polymer species. We also approximate for the sake of simplicity that ε̅_0_ ≅ ε_0_. Both approximations have a minor effect on the molecular orientation because (i) at small extension, the orientation is dominated by the adsorption, regardless of the initial extension or relaxation time, and (ii) at large extension, the relative effect of the small initial extension or relaxation time is negligible. Accordingly, eq 5 (2D) becomes identical in form to eq 4 (3D), except for the lower value of the critical strain rate in 2D (eq 6), as seen in Figure 2 in the left-shift from the solid curves to the long-dash curves ().

### 3.2 Chain Extension in a Polymer Blend

Together with the jet strain rate *s*, the chain relaxation time τ and extension at rest ε_0_ are the variables that determine the degree of chain extension. A higher strain rate results in a higher tension and extension. Similarly, a long relaxation time slows down stress relieving, resulting in a higher extension. In the case of a polymer blend (Figure 1d) comprising polymer species of disparate degrees of polymerization *N* and Kuhn length *b*, the relaxation times and initial extensions will be dissimilar as well (Table 1). As a result, the degree of extension of the two-polymer species and consequently their molecular orientation vary widely. PVP experiences a stretch transition at a much lower strain rate compared to MEH-PPV (Figure 2).

**Table 1:** Polymer, Solution, and Network Properties^a^

species^b^	polymer^c^					solution			network^d^		
	*b* [nm]	*N*_e1_	*M*_0_ [Da]	*M*_W_ [kDa]	*N* [*M*_W_/*M*_0_]	*c* [g L^–1^]	ρ [g cm^–3^]	φ [*c*/ρ]	ε_0_	τ [ms]	*s*_c_ [s^–1^]
PVP	1.76	15	766	1300	1697	30	1.25	0.023	0.034	0.133	2.3 × 10^4^
MEH-PPV	6.18	1	2764	200 ± 50	72	30	1.25	0.023	0.130	0.010	2.9 × 10^5^

a Notes: symbols: *b* Kuhn segment length, *N*_e1_ strand segments in melt, *M*_0_ segment molar mass, *M*_W_ polymer molar mass, *N* segments in the chain, *c* polymer concentration, ρ polymer mass density, φ polymer volume fraction, ε_0_ initial chain extension, τ chain relaxation time, and *s*_c_ critical strain rate.

b Polymer blend: poly[2-methoxy-5-(2-ethylhexyloxy)-1,4-phenylenevinylene] (MEH-PPV); polyvinylpyrrolidone (PVP). Solvent: chloroform/dimethyl sulfoxide (CHCl_3_/DMSO) 9:1 v/v, with viscosity *η*_s_ = 0.75 × 10^–3^ Pa s, assumed θ-solvent.

c Polymer data: based on ref (54) and estimation.

d Network data: using the relations in Supporting Information Section S2 (θ-solvent), with the overall polymer volume fraction φ = 0.046. To obtain the parameters for a good solvent for both species, the values in (d) have to be rescaled as follows: ε_0_ should be multiplied by 1.48, τ by 8.29, and *s*_c_ by 0.12.

In the network, each of the two polymers interacts with its own species, as well as with the other species (Figure 1d). To capture the topological interactions between the two polymers, which may affect the critical strain rates and the initial extension, we used the overall polymer concentration in the blend (volume fraction of φ = 0.046, Table 1), rather than the concentration of each polymer separately (φ = 0.023). The polymer network values in Table 1 are calculated for a θ-solvent, for which chains have an ideal conformation (Table 1). Specifically for the solution used in our experiments, the predominant solvent, chloroform, is a good solvent for both MEH-PPV and PVP,^55,56^ and the network values can be adjusted accordingly as noted in Table 1; the main effect is a uniform left-shift of the plots in Figures 2^3^,4. That said, the solvent quality has a negligible effect on the relative strain rate *s*/*s*_c_, which determines the degree of chain extension and molecular orientation (see Section 3.6).

**Figure 3:**
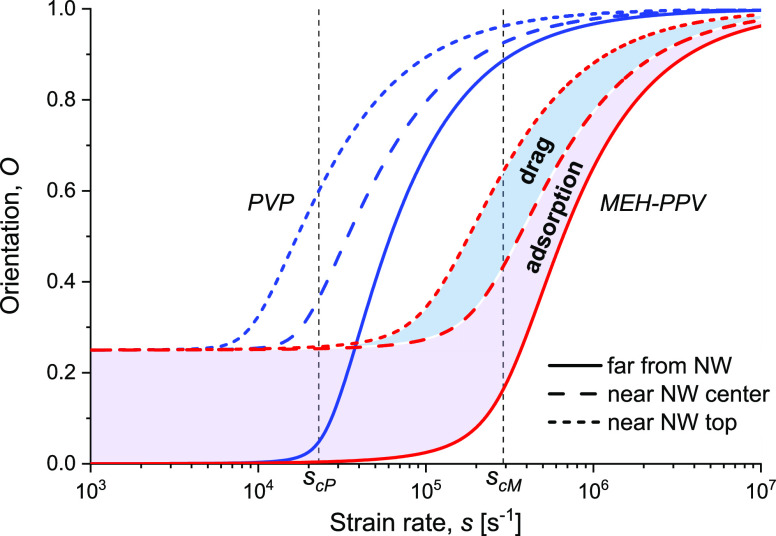
Molecular orientation in an electrospun polymer blend. Log-linear plot of the total orientation *O* [eqs 9, 10, and 12] for each polymer species, depicted as a function of the electrospinning strain rate *s*, for the polymer blend specified in Table 1. The critical strain rates *s*_cP_ (PVP) and *s*_cM_ (MEH-PPV) are indicated. The unconfined orientations are depicted by solid lines; the region marked as “adsorption” denotes orientations close to the NW center; the region marked as “drag” denotes orientations close to the NW top, where both adsorption and flow drag are significant.

**Figure 4:**
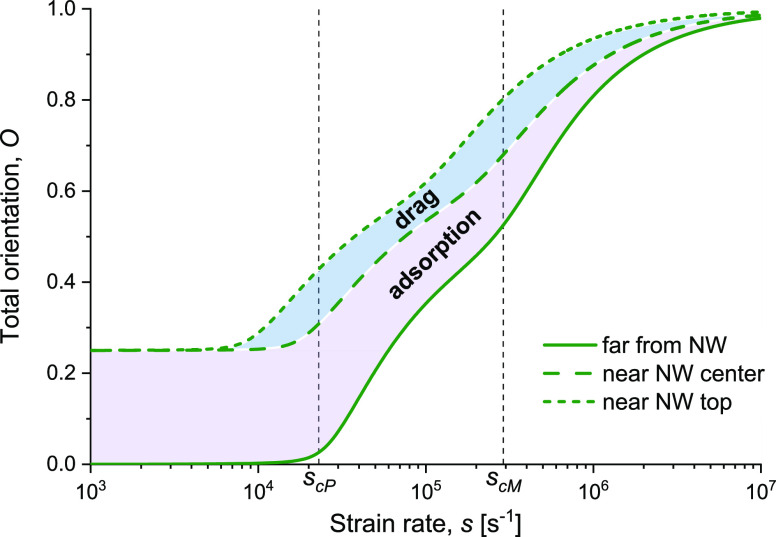
Total molecular orientation in an electrospun polymer blend. Log-linear plot of the total orientation *O* (eq 11) for both polymer species, depicted as a function of the electrospinning strain rate *s*, for the polymer blend specified in Table 1. The critical strain rates *s*_cP_ (PVP) and *s*_cM_ (MEH-PPV) are indicated. The unconfined orientations are depicted by a solid line; the region marked as “adsorption” denotes orientations close to the NW center; the region marked as “drag” denotes orientations close to the NW top, where both adsorption and flow drag are significant.

The two-polymer species differ significantly in their conformation. While the backbone of a PVP chain is composed of a sequence of single covalent bonds and is therefore flexible, the MEH-PPV chain is composed of alternating single and double covalent bonds (conjugated bonding), making it highly rigid in bending. However, inherent bonding defects in the MEH-PPV partially substitute rigid conjugated links with flexible tetrahedral links, making the polymer chain semiflexible with long rigid segments (Kuhn monomers).^8,9^ The typical segment length is between 1 and 10 beads (5 on average) between defects, each bead having a diameter of 1.2 nm (Table 1). The extended lengths of the chains are *L*_max_ = *bN* ≅ 3000 and 450 nm for the PVP and MEH-PPV, respectively, whereas their free end-to-end distances in a θ-solvent are *R*_0_ = *bN*^1/2^ ≅ 70 and 50 nm, respectively; hence, the conjugated polymer is nearly as large as the flexible polymer, even though its molar mass is about 6.5 times smaller. The number density of MEH-PPV chains is 6.5 times larger than the PVP density, that is, on average 6.5 MEH-PPV chains for each PVP chain (Figure 1d). Similarly, the relaxation time, which scales as τ ∝ *b*^3^*N*^2^ (see Supporting Information Section S2), is about 13 times shorter in MEH-PPV than in PVP; hence, the MEH-PPV critical strain rate, *s*_cM_, is about 13 times higher than that of PVP, *s*_cP_, as seen in Figure 2.

In terms of the relative extension, the PVP/MEH-PPV polymer blend has three different regions: (a) the region below the critical strain rate of PVP, *s* < *s*_cP_, where both polymers are only negligibly stretched; (b) the region between the critical strain rates of PVP and MEH-PPV, *s*_cP_ < *s* < *s*_cM_, where PVP is stretched significantly, whereas MEH-PPV is not; and (c) the region above the critical strain rate of MEH-PPV, *s* > *s*_cM_, where both polymers are stretched significantly. In the latter region, the high strain rate leads to rapid separation between entanglement nodes, resulting in the disentangling of chains from the network. While the PVP long chains may still be entangled in a network, maintaining the integrity and contiguousness of the network, the MEH-PPV chains may be fully disentangled from the network because of their shorter length and lesser extension.^57^ For example, as the primitive length of the PVP in that region is about an order of magnitude longer than that of MEH-PPV, at the instant MEH-PPV disentangles (2 nodes at its edges), PVP is still entangled in 10 nodes. The MEH-PPV chains keep moving with the network and are extended by the jet strain rate as expressed in the model in eq 4.

### 3.3 Molecular Orientation and Adsorption

Thus far, we have expressed the relative extension of polymer chains, both those far from a boundary and those near it, in terms of the relative strain rate of the electrospinning jet. We now turn to estimating the molecular orientation associated with the extended conformation of chains. A linear polymer chain can be expressed as a sequence of *N* successive Kuhn monomers of length *b*, placed on a 3D Cartesian lattice.^32^ Given a force *F_i_* acting in direction *i*, the energy associated with a monomer aligned in that direction is *F_i_b*. The corresponding statistical Boltzmann factor is e^–*F_i_b*/*k*_B_*T*^, where *k*_B_ is the Boltzmann constant, and *T* is the absolute temperature. The exponent *f_i_* = *F_i_b*/*k*_B_*T* is the normalized tension force in direction *i*. Thus, the probability that a monomer will align in direction *i* is given by^10^
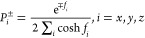
7where the + and – signs indicate the positive and negative directions, respectively. The denominator ensures that the sum of all the alignment probabilities equals 1. In the jet longitudinal direction (*y* axis), the extensional force is *f_y_* = *f*(ε), whereas in the lateral directions, the force is that of a network at rest, *f_x_* = *f_z_* = *f*(ε_0_). We ignore the effect of radial compression associated with longitudinal extension because it can be considered as negligible (see Supporting Information Section S3).

The relative extension is given by ε = ε_*y*_ = *P*_*y*_^+^ – *P*_*y*_^–^, and the initial extension is obtained by substituting *f_y_* = *f*(ε_0_). Using Hermans’ orientation parameter, , the orientation is given by adding up all *N* segments of a chain, , where θ_*i*_^±^ is the angle of a segment with respect to the longitudinal axis. As in the Cartesian lattice  and θ_*y*_^±^ = 0, we obtain
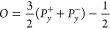
8

Using eqs 7 and 8 and solving for the orientation in terms of the extension (see details in Supporting Information Section S3), one has
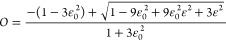
9where ε_0_ is given in Supporting Information Section S2 and ε by eq 4. Scaling approximations at small ε_0_ yield *O* ≈ ε_0_^2^*s*τ for low *s*τ and *O* ≈ 1 – 9/2*s*τ for high *s*τ (see Supporting Information Section S4).

Near a boundary, such as the surface of an NW, monomer alignment perpendicular to the surface is precluded due to adsorption of monomers. Since the NWs are assumed to be aligned with the flow (see experimental evidence in Section 4.1), the adsorption occurs in one of the lateral directions, say *z*, and the probability of alignment in that direction is null, that is *P*_*z*_^±^ = 0, and the terms containing *f_z_* are removed from eq 7. This reduces the overall sum of probabilities, increasing the probabilities of alignment in the *x* and *y* directions. The orientation in terms of the extension for this case is (see details in Supporting Information Section S3)
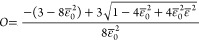
10where ε̅_0_ ≅ ε_0_ is given in Supporting Information Section S2 and ε̅ by eq 5. Both eqs 9 and 10 are independent of the explicit values of *f_i_*. Scaling approximations at small ε_0_ yield *O̅* ≈ (1 + 3ε̅_0_^2^*s*τ̅)/4 for low *s*τ and *O̅* ≈ 1 – 3/*s*τ̅ for high *s*τ (see Supporting Information Section S4).

The unconfined 3D orientations and the confined 2D orientations are depicted in Figure 3. Note that the 2D extensions and orientations constitute upper limits in the vicinity of a boundary, as they are based on the expansive assumption of full chain adsorption in one of the lateral directions, a condition that might apply only partially (for example, slightly away from a boundary or in weak adsorption). However, as we are addressing general behavioral trends, this condition seems acceptable. Useful scaling approximations for the extension and orientation expressions near a boundary and away from it, expressed in terms of the relative strain rate *s*/*s*_c_, are given in Supporting Information Section S4.

While analyzing solid nanofibers to measure alignment by SNOM (see Section 4), it is possible to separate the orientation of MEH-PPV from the total orientation by using a laser beam with a wavelength in the absorption band of the conjugated polymer component (473 nm, a wavelength in which PVP has negligible absorption—measured fraction of light that is transmitted by a PVP film at 473 nm >99%). If a concomitant study of the PVP and MEH-PPV molecular orientation is desired, it could be probed by using polarized infrared scanning microscopy techniques, such as scattering-type SNOM and atomic force microscopy-infrared spectroscopy, through spectroscopic investigation of vibrational modes characteristic of either PVP or MEH-PPV.^58,59^

In case the total orientation is desired, that is, the combined contribution to orientation of all polymers in a blend, Hermans’ parameter can be expressed as a weighted summation of the alignment probabilities of each polymer *j*
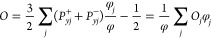
11where φ_*j*_ is the volume fraction of polymer *j*, φ = ∑_*j*_φ_*j*_ is the total solution concentration, and *O_j_* is the orientation of polymer *j* obtained by eq 9 or 10. The expression on the right was obtained by using the equality in eq 8. This simple rule of mixture is depicted in Figure 4 for the two-polymer blend of our study. Note that although the PVP long chains reach a significant orientation at a relatively low strain rate, the high degree of the overall molecular orientation is achieved only after the short chains of the MEH-PPV are highly oriented as well at a much higher strain.

### 3.4 Strain Rate and Hydrodynamic Drag

An NW in the electrospinning flow moves at the same velocity of the solution near the NW center. This can be verified by comparing the hydrodynamic and acceleration forces acting on the NW: (i) the hydrodynamic force scales as η*Vl*, where η is the solution viscosity (typically 0.1 to 10 Pa s in semidilute entangled solutions), *V* is the solution velocity, and *l* is the NW half-length; (ii) the acceleration force scales as ρ*d*^2^*lV̇*, where ρ = 7.2 g cm^–3^ is the NW density, *d* is its diameter, and *V̇* = *V*∇*V* is the solution acceleration; the ratio between the forces is η/ρ*d*^2^∇*V*∼10^6^, meaning that the hydrodynamics is predominant.

**Figure 5:**
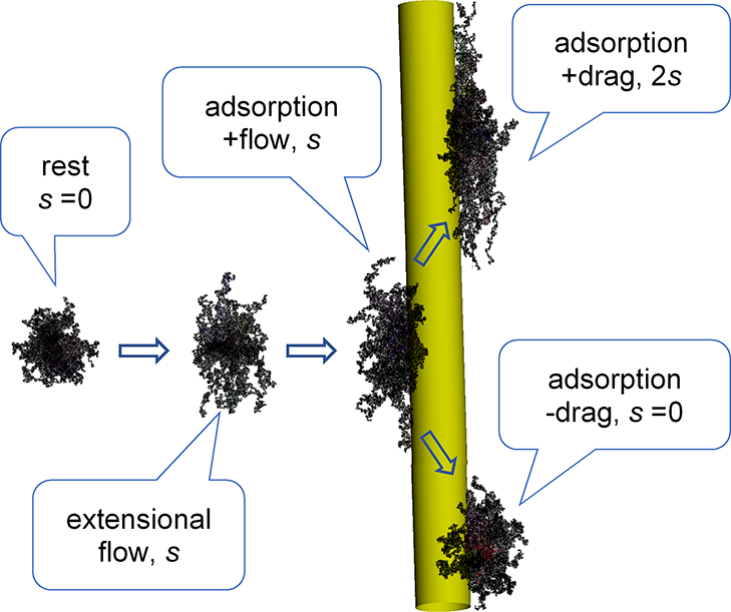
Illustration of polymer adsorption and drag nearby an NW. Simulation of 50 free chains of *N* = 1700 monomers, near an NW and far from it. The strain rate of the free extensional flow is *s* = 10^6^ s^–1^. The coil shape of chains at rest is shown for reference on the left.

The hydrodynamic drag exerted by an NW on the flow affects the strain rate locally. The velocity of the NW along its length is constant, whereas the velocity of the solution with respect to the NW varies with the downstream distance *y* from the NW center, *v* = *sy* (Figure 1e). The maximal velocity difference is at the NW ends, *v*_max_ = *sl*, of order 1–10^2^ mm s^–1^. Thus, at the NW lower half, the solution velocity is faster than the NW velocity, so that the NW drags the solution upward, creating a negative local strain rate of −d*v*/d*y* = –*s*. Conversely, at the NW upper half, the solution velocity is slower than the NW velocity, so that the NW drags the solution downward, creating a positive local strain rate of d*v*/d*y* = *s*. Combined with the jet strain rate *s*, the strain rate at the NW upper end is 2*s* and at its lower end it is 0. The dragging effect at the NW top, combined with the effect of adsorption, is depicted by the short-dash curves in Figures 2–4, left-shifted by a factor of 3 with respect to the 3D curves (3/2 factor due to adsorption and 2 factor due to drag). The combined effect of the extensional flow and the adsorption and positive/negative drag near an NW is illustrated in Figure 5.

This prediction is an upper limit for the change in the strain rate, as the velocity difference between the chains adjacent to the NW and those far from it occurs across the fluid boundary layer. If the boundary layer is larger than the scale of a typical chain, the chain will sense only a fraction of the velocity difference and hence a fraction of the strain rate change. The boundary layer thickness is inversely related to the fluid velocity, and therefore, a thicker layer tends to form near the NW center, resulting in a lower strain rate, and a thinner layer forms near the ends, resulting in a higher strain rate. As a complete fluid analysis is beyond the scope of the current study, we use the following simplified upper-bound expression for the strain rate along an NW boundary
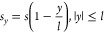
12assuming linear variation with the longitudinal distance *y* from the NW midpoint, such that the value of *s_y_* is *s* at the midpoint (*y* = 0), 2*s* at the top (*y* = – *l*), and 0 at the bottom (*y* = *l*).

### 3.5 Orientation Mapping

**Figure 6:**
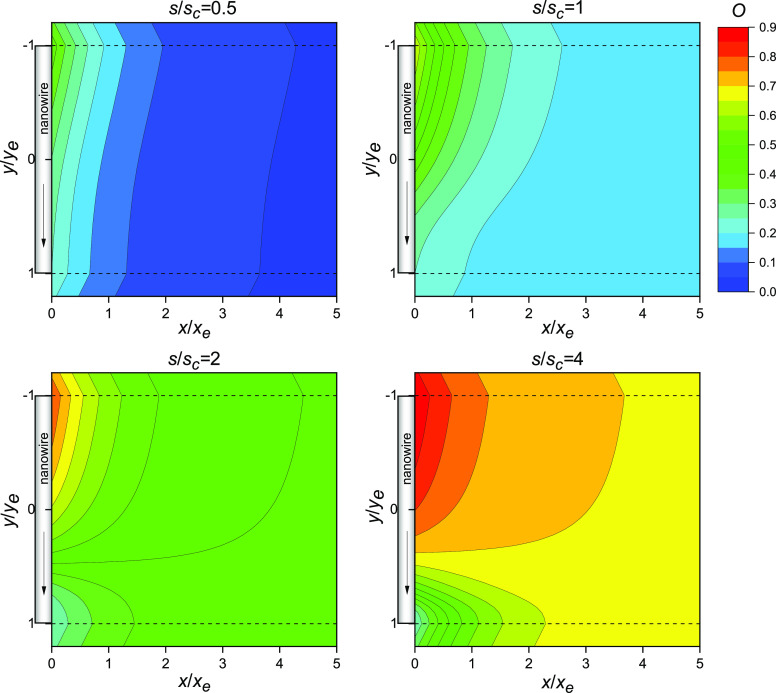
Molecular orientation maps near an NW. Contour maps of the orientation mapping function (eq 13) in the *x*, *y* space, where *x* is the radial distance from the NW boundary and *y* is the axial downstream distance from the NW midpoint. *x* is normalized by the characteristic orientation extinction distance *x*_e_, and *y* is normalized by the extinction distance *y*_e_. The maps are depicted for four values of the relative strain rate *s*/*s*_c_, all referring to the same color scale. The flow direction is indicated by arrows. The longitudinal extinction distance is *y*_e_ = *l*, the NW half-length. The initial extension is that of MEH-PPV, ε_0_ = 0.130.

To visualize the orientation near an NW, the orientation is mapped in the *x*, *y* space, where *x* is the radial distance from the NW boundary and *y* is the axial downstream distance from the NW midpoint (Figure 6). Two assumptions are made: (a) the change in the strain rate due to hydrodynamic drag along the NW boundary varies proportionally to the axial distance *y* (eq 12); and (b) the orientation effect produced by both adsorption and drag decays exponentially as a function of the radial distance *x*, with a characteristic extinction distance *x*_e_; similarly, the orientation decays exponentially as a function of the longitudinal distance beyond the ends of the NW, |*y*| – *l*, with a characteristic extinction distance *y*_e_. These assumptions lead to the following orientation mapping, expressed in terms of the relative strain rates
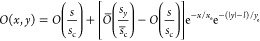
13*s*/*s*_c_ is an independent variable, whereas  using eq 6, where *s_y_* is given in terms of *s* by eq 12 when |*y*| ≤ *l*. Moreover, *s_y_* is taken as constant above and below the NW, such that *s_y_* = 2*s* when *y* < – *l*, and *s_y_* = 0 when *y* > *l*.

The longitudinal decay (second exponential function) applies only when |*y*| > *l* and is otherwise equal to 1. The functions of *O* used in this equation are obtained from eqs 9 and 10. As required, this mapping function returns the unperturbed orientation when *x* ≫ *x*_e_ and/or |*y*| – *l* ≫ *y*_e_ and the adsorption + drag orientation when *x* → 0 and |*y*| ≤ *l*. As the variables *x*, *y*, and *s* are normalized by the corresponding parameters *x*_e_, *y*_e_, and *s*_c_, respectively, eq 13 is universal, with only a single physical parameter, i.e., the initial chain extension ε_0_.

The radial extinction distance *x*_e_ could possibly be in the order of the polymer network correlation length *b*/φ,^32^ or the NW half-length *l*, both of order 10^2^ nm. However, *x*_e_ is not necessarily the same for adsorption and drag, and in such a case, eq 13 would have to be generalized by associating a different *x*_e_ with each contribution. For simplicity’s sake, the following maps assume the same extinction distance. The longitudinal extinction distance *y*_e_ could be in the order of the NW length or a fraction of it.

The orientation mapping function *O*(*x*, *y*) is depicted in Figure 6 for four values of the relative strain rate *s*/*s*_c_, for the initial extension of MEH-PPV from Table 1. We see that at strain rates below or at the critical strain rate (the top two maps), the orientation is generally low; it is higher near the NW, where it is dominated by adsorption, than far from the NW. However, at strain rates above the critical strain rate (the two bottom maps), the orientation is generally high, and the dominant effects are the jet strain rate and the hydrodynamic drag. The orientation is nonuniform along the NW, with a high orientation at its top and a low orientation at its bottom. Furthermore, the orientation at the bottom of the NW is lower than the orientation far from the NW, splitting the map into two regions, above and below the far orientation.

As noted, beyond the NW ends, these effects are assumed to decay exponentially. Thus, at low strain rates, the orientation dominated by adsorption decreases gradually beyond both NW ends to the low value of the unperturbed flow. By contrast, at high stain rates, above the NW top end, the orientation enhanced by dragging decreases gradually toward that of the unperturbed flow, whereas below the bottom end, the orientation reduced by restraining increases gradually toward that of the unperturbed flow. One should keep in mind that these maps represent upper-limit orientation values and should therefore be regarded merely as trends to be supported by experiments (see the Experimental Analysis Section, Section 4).

An additional feature, unrelated to the presence of NWs and therefore not shown in the maps, is the anisotropy of the molecular orientation across the fiber (radial direction). The jet longitudinal strain rate *s* is accompanied by a radial strain rate of −*s*/2, exerting radial compression on the polymer chains.^49^ As a result, the solution near the jet center is more polymer-rich (higher concentration, φ), raising both ε_0_ and τ (equations in Supporting Information Section S2) and consequently the extension and longitudinal molecular orientation. At the same time, the polymer network near the nanofiber boundary is extended in the radial direction, causing a radial molecular orientation. These effects are substantiated theoretically^10,60−62^ as well as experimentally^8,9,52^ by X-ray imaging of jets, near-field optical imaging of nanofibers, and AFM elastic modulus measurements at nanofiber cross sections. They can also be observed by the optical measurements of nanofibers in the current study (see Section 4). Thus, when NWs are included in the solution, their effect on orientation should vary depending on whether they are in the jet core (this study) or near the jet boundary.

### 3.6 Effect of Electrospinning Conditions

The relative strain rate and, consequently, the degree of chain extension and molecular orientation depend on the electrospinning conditions. A complete analysis of all parameters involved in the electrospinning process is highly complex. We therefore focus on the readily tunable parameters—the jet exit velocity *v*_0_ (feed rate), the electric field intensity *E*, and the solution concentration φ. The parametric dependence of the relative strain rate scales as  (θ-solvent).^10^ The other parameters include the monomer length, injector internal diameter, solution electric conductivity, solvent viscosity, and degree of polymerization. The exponents of φ for a good solvent (−2.96) and for a θ-solvent (−2.89) are similar, implying low dependence on solvent quality.

Using a scaling approximation for high strain rates (Supporting Information Section S4), the orientation far from an NW scales as
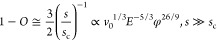
14The orientation near an NW scales in the same way, except for an additional prefactor of  (eqs 6 and 12). The quantity 1 – *O* signifies the distance of the orientation from the possible maximum (*O* = 1). At low strain rates, the orientation scales as *O* ∝ s/*s*_c_. We see that the orientation increases when the electric field is increased and when the feed rate or concentration is decreased. The different power exponents indicate that coarse tuning can be conducted by modifying the concentration, and moderate tuning can be conducted by modifying the electric field, whereas fine tuning is achieved by modifying the feed rate. It should be noted that, in order to ensure jet formation, continuity, and contiguousness and to prevent jet breaking, the electrospinning process parameters should be kept within the physical bounds of the process by remaining within the combined working ranges of the feedrate, electric field, and solution concentration.^57^

The relationship presented above does not provide an accurate value of the relative strain rate *s*/*s*_c_, as both *s* and *s*_c_ are obtained by scaling analysis, which yields only trends. One approach to determining the stretching regime is to track the evolution of the orientation for a range of strain rates, tuned by a single parameter. A plot of *O* vs that parameter, similar to Figure 3, would reveal the orientation evolution and the stretching domains. Another approach to estimating *s*/*s*_c_ is to compare the modeling and experimental trends, as we do in the following section. Overall, the jet strain rate is dominated by PVP because of its long chains and high degree of entanglement. Consequently, the fiber diameter is also dominated by PVP. This is a desirable outcome, as it yields fibers with diameters of the order of a few microns, suitable for SNOM characterization.

## 4 Experimental Analysis

### 4.1 Electrospun Fiber Properties

**Figure 7:**
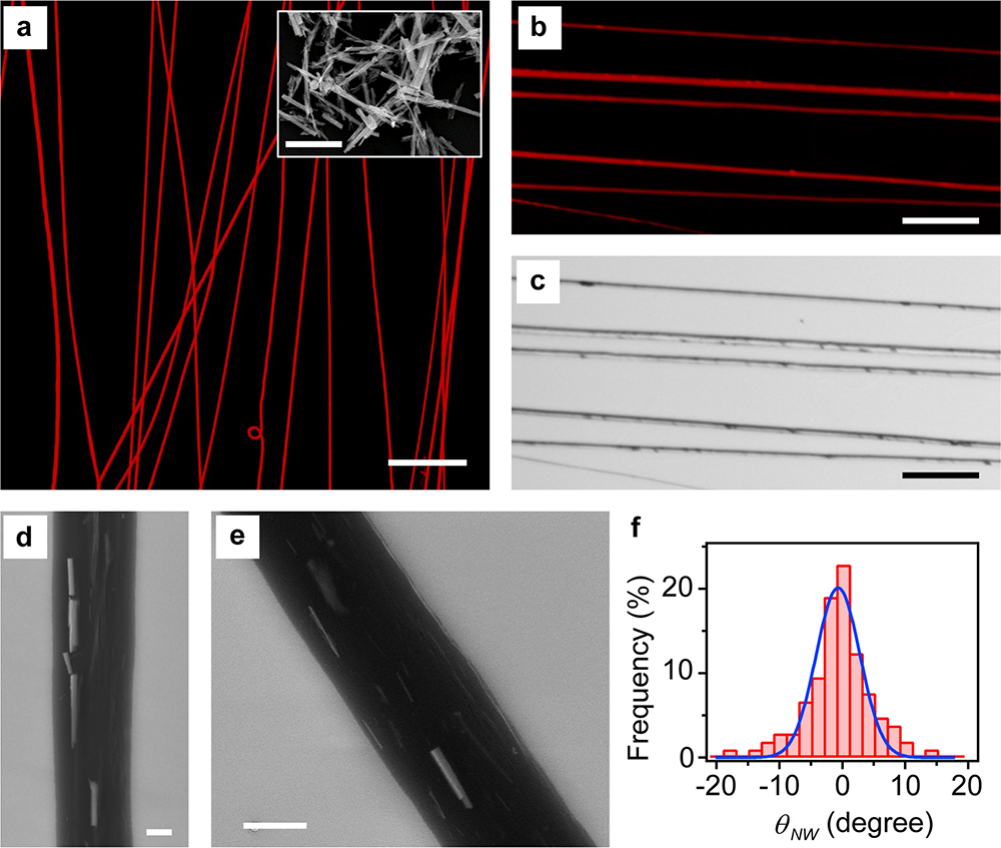
Nanofibers’ fluorescence and transmission. (a,b) Fluorescence confocal micrographs of PVP/MEH-PPV/WO_3_ electrospun nanofibers. Inset in (a): SEM micrograph of WO_3_ NWs, with scale bar = 2 μm. (c) Map of the intensity of the excitation laser transmitted through the sample and acquired simultaneously with the fluorescence map shown in (b). Scale bars: (a) 100 μm, (b,c) 25 μm. (d,e) SEM micrographs of single PVP/MEH-PPV/WO_3_ fibers showing embedded NWs at the fiber surface. Scale bars: (d) 500 nm, (e) 1 μm. (f) Distribution of the orientation angles, θ_NW_, of the WO_3_ NWs observed by SEM on the fibers’ surface, with respect to the fiber’s long axis. 0° corresponds to NWs aligned along the fiber length.

Figure 7a,c shows exemplary confocal microscopy images of nanocomposite electrospun fibers made of WO_3_ NWs (average length 600 nm, max length < 2 μm, average diameter 50 nm, inset of Figure 7a) embedded in a PVP/MEH-PPV matrix.

The fluorescence micrographs (Figure 7a,b) show the characteristic emission of the conjugated component, MEH-PPV, in the visible range. The emission is fairly uniform along the fiber length, while the incorporated NWs appear as dark scattering spots in the maps of the intensity of the excitation laser transmitted by the fibers (Figure 7c). Figure 7d,e shows scanning electron microscopy micrographs of the composite nanofibers, with some NWs clearly visible on the fiber surface. These NWs are well aligned along the fiber length (Figure 7f), their long axis forming on average an angle, θ_NW_ (defined as the angle formed by the long axes of the NW and nanofiber, respectively), close to zero, with a standard deviation of 5 degrees.

### 4.2 Molecular and Optical Anisotropy

**Figure 8:**
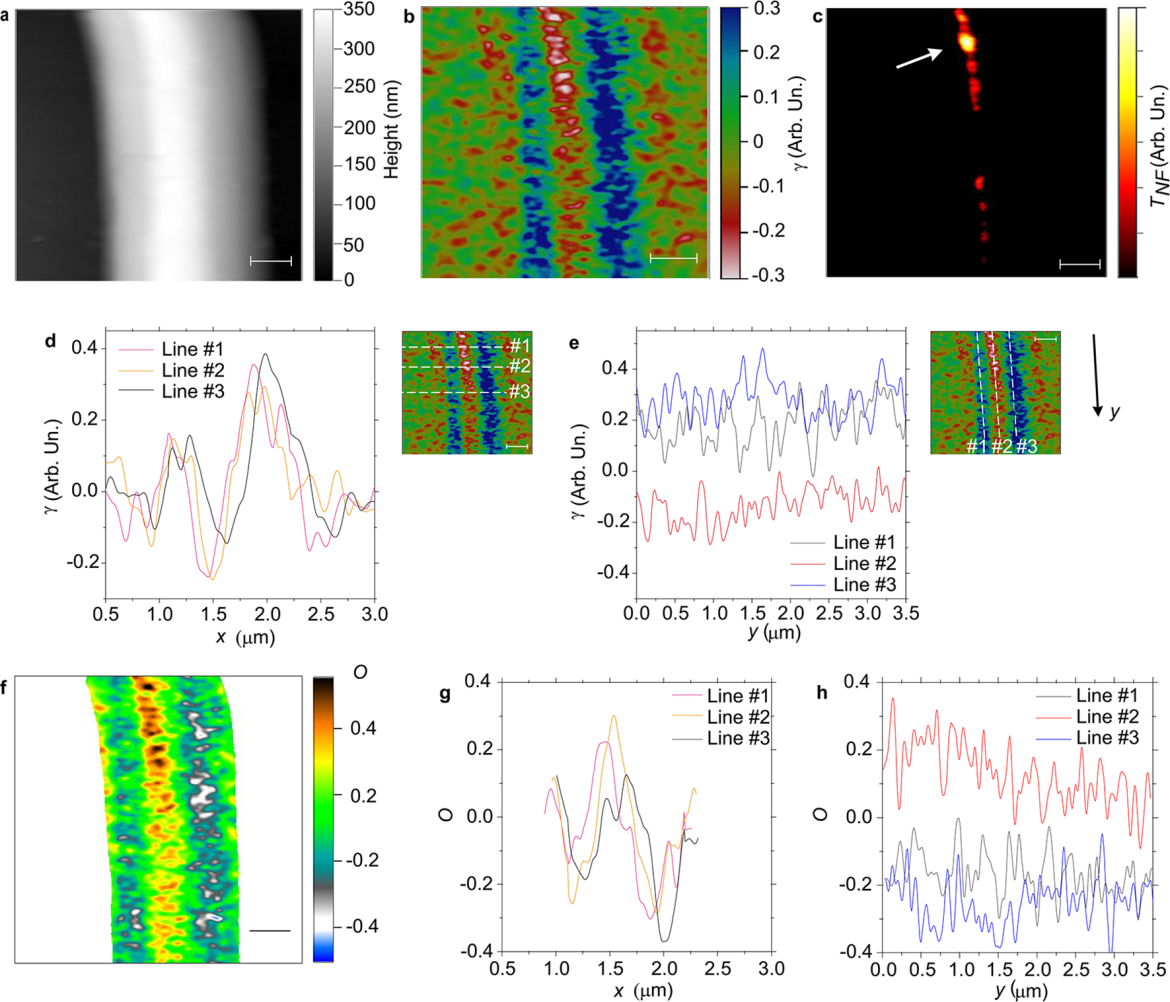
Nanofibers’ molecular and optical anisotropy. (a) Topography map of a single PVP/MEH-PPV/WO_3_ electrospun fiber. (b,c) Corresponding maps of the (b) dichroic ratio, γ, and of the (c) near-field excited optical extinction signal, *T*_NF_. Scale bars: 500 nm. The arrow in (c) highlights the region with WO_3_ NWs. (d,e) Line profile analysis of γ displaying the cross sections along the (d) horizontal, *x*, and (e) vertical, *y* (positive downward), dashed segments shown in the insets, respectively. Negative values of γ are indicative of alignment of the MEH-PPV polymer chains along the fiber length. Inset scale bars in (d) and (e): 500 nm. (f) Map of Herman’s orientation parameter *O*, and (g) horizontal and (h) vertical line profiles along the dashed segments shown in the insets of (d) and (e), calculated for the fiber shown in (a–c).

Figure 8 shows an example of the results of polarization-modulation SNOM (see the Experimental Methods Section for details), where areas with NWs at a low density close to the fiber central axis are selected, in order to decrease possible contributions to the measured signals due to light scattering from NWs and due to relevant variations of the fiber topography. An additional example is shown in the Supporting Information (Figure S1).

In particular, Figure 8b shows a map of the linear dichroism, γ, evidencing a nontrivial local variation, especially nearby the WO_3_ NWs (top central area of the map of the extinction, Figure 8c, highlighted by the arrow). The map of the local fiber thickness (topography, Figure 8a) is acquired simultaneously with the optical signal. The dichroic ratio in a region without NWs is seen in the lower part of Figure 8b: an area with negative values of γ, corresponding to MEH-PPV macromolecules mainly aligned along the fiber length, and an area with molecules preferentially oriented along the radial direction are present, as found also in pristine MEH-PPV electrospun fibers.^8,9^ A spatial variation of the internal architecture has been reported also for other fiber species, such as electrospun polycarbonate nanofibers.^63^

The presence of NWs impacts the nanoscale local arrangement of the MEH-PPV macromolecules and determines, on average, a local spatial variation of the dichroic ratio. In fact, the presence of the NW leads to an average increase of the absolute value of γ (by a factor of about 2) at the fiber central axis, with MEH-PPV macromolecules aligned along the fiber length, as can be observed by comparing the line profiles of γ along directions parallel and perpendicular to the NW length (Figure 8d,e, respectively). Specifically, in Figure 8d, the negative peaks of γ at the nanofiber center (*x* ≅ 1.5) are lower in the NW vicinity (lines 1 and 2, pink and orange) than further away from it (line 3, black) by a factor of 1.7. In Figure 8e, γ is lower on average by a factor of 2.3 in the NW vicinity (line 2, red, left part) compared to the region further from it (right part of line 2). The orientation parameter calculated from the measured values of γ is shown in Figure 8f–h. These values are calculated by the relation^58^, where  and α is the angle formed by the direction of the fiber length and a reference direction that is experimentally determined (α is about 25 degrees). Similar results are shown in Figure S2 in the Supporting Information. This is indicative of an average increase of the alignment of the MEH-PPV macromolecules caused by the NWs.

### 4.3 Comparison to Theory and Discussion

**Figure 9:**
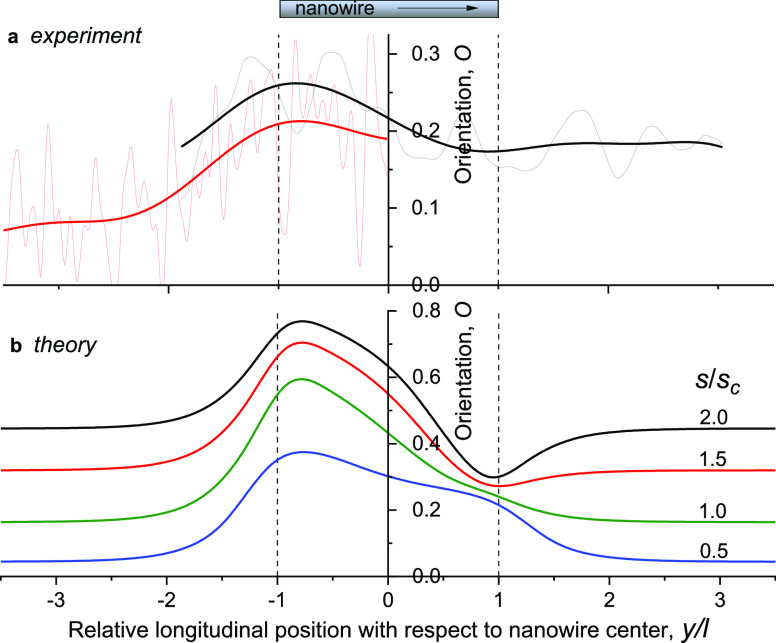
Comparison of the experiment to theory. (a) Molecular orientation (Herman’s parameter) derived from the dichroic ratio measured by polarized microscopy of the core of a PVP/MEH-PPV/WO_3_ electrospun fiber. Data from Figure 8e and Supporting Information Figure S2a are smoothed by FFT filtering (red and black curves, respectively). The NW position is depicted above, with an arrow indicating the flow direction (this implies that the flow direction in Figure 8b is along the negative *y* axis, whereas in Figure S2a of the Supporting Information, it is along the positive *y* axis). (b) Molecular orientation along an NW centerline, based on the theoretical mapping in eq 13 at radial position *x* = 0, depicted for four values of the relative strain rate *s*/*s*_c_ and smoothed. The same conditions as in Figure 6 apply.

The results obtained from the theoretical model we developed are in line with the experimental findings. Let us start with the example of Figure 8e,h (the red curve in Figure 9a). Far from the NW, the dichroic ratio at the fiber core has an absolute value of about 0.1, corresponding to an orientation parameter of about 0.1 (Figure 9a, left side), an indication of significant polymer network stretching and molecular orientation. This implies that the strain rate *s* of the electrospinning jet is about the same or slightly exceeds the critical strain rate *s*_c_ of the MEH-PPV polymer, expressed, for example, by the theoretical curve of *s*/*s*_c_ = 1 in Figure 9b. The corresponding theoretical molecular orientation far from an NW is about 0.2 in this strain rate condition (*y*/*l* ≪ – 1).

Near the top end of the NW (*y*/*l* = – 1), both the experimental and theoretical molecular orientations rise sharply above the values far from the NW by a factor of about 2, owing to the combined effect of the higher drag at the fiber top and the adsorption of polymer chains near an object. Toward the NW midpoint (*y*/*l* = 0), both the experimental and theoretical molecular orientations decrease moderately as a result of the diminishing strain rate and drag but are still higher than values obtained far from the NW. At the midpoint, the drag effect vanishes, whereas the adsorption effect still enhances the orientation.

These trends can be observed in the example of the Supporting Information, Figure S2a (the black curve in Figure 9a) as well. Far from the NW (*y*/*l* ≫ 1), the orientation in this example is about 0.2, implying a higher relative strain rate, likely expressed by the theoretical curve of *s*/*s*_c_ = 1.5 in Figure 9b. From the theoretical plot (Figure 9b) we expect a gradual convergence to the values far from the NW, with a possible negative peak around the NW bottom (*y*/*l* = 1), where the low strain rate and drag counteract the adsorption. This is indeed observed in the black curve in Figure 9a. At low relative strain rates (*s*/*s*_c_ ≤ 1), there is no negative peak in that region because the effect of adsorption is far greater than that of drag.

As already noted, the theoretical analysis represents upper-limit orientation values. We find evidence of this in the value of Herman’s orientation parameter derived from the dichroic ratio in Figures 8e and S2b. The peak experimental value is about 0.3, whereas the corresponding theoretical values are about twice as high. The major reason for this difference is likely the partial relaxation of the polymer network after the jet reaches the collector, when further stretching ceases, while the fiber is not yet fully solidified.

## 5 Conclusions

The molecular orientation of an electrospun MEH-PPV conjugated polymer, blended with a PVP polymer, is studied theoretically, focusing on the extension caused by electrospinning and the local adsorption and drag induced by WO_3_ NWs. Our aim is to rationalize and ultimately enhance the orientation of conjugated polymer chains, a condition critically important for improving the polarization degree of the light emitted by the hybrid nanofibers. It is shown that the polymers undergo a stretch transition at a critical jet strain rate, leading to the corresponding high molecular orientation. Adsorption of chains near the boundary of an NW causes further orientation along the jet axial direction. Furthermore, the hydrodynamic drag induced by an NW on the flow affects the local strain rate, with matching variation in local orientations. These effects are mapped and tuned to the electrospinning conditions.

These predictions are supported by experimental evidence derived from electrospun MEH-PPV/PVP/WO_3_ nanofibers, imaged by polarized SNOM. The mapping of the optical dichroic ratio of the MEH-PPV demonstrates the molecular anisotropy caused by the NWs. These measurements also unveil an increased molecular alignment of the polymer molecules along the fiber axis in the vicinity of the NWs, supporting the predicted effect of adsorption. Furthermore, the dichroic ratio is not distributed uniformly along the NW length, indicating the predicted effect of hydrodynamic drag.

These synergetic results demonstrate that the mixing of WO_3_ NWs with the MEH-PPV conjugated polymer could be advantageous for the optical properties of the nanofiber, potentially improving its ability to transport light excitations as a result of molecular alignment. Further research is envisioned using higher resolution optical scanning of nanofibers to map the molecular orientation, characterization of the optical properties of these fibers, and tuning of the electrospinning materials and process conditions.

## Supporting Information Available

Calculation of the stretching equation and force function; calculation of chain relaxation time and initial extension; calculation of chain elongation and orientation; scaling approximations for chain elongation and orientation; and additional experimental data of molecular and optical anisotropy (PDF)
