# Supplementary material for: WO3 Nanowires Enhance Molecular Alignment and Optical Anisotropy in Electrospun Nanocomposite Fibers: Implications for Hybrid Light-Emitting Systems
Source: ACS Appl Nano Mater. 2022 Mar 9;5(3):3654–66. doi: 10.1021/acsanm.1c04110 (PMC8961744; doi:10.1021/acsanm.1c04110)
Supplement: Supplementary file 1 — an1c04110_si_001.pdf [file an1c04110_si_001.pdf]

Supporting Information

WO_3 Nanowires Enhance Molecular Alignment and Optical Anisotropy in Electrospun Nanocomposite Fibers: Implications for Hybrid Light-Emitting Systems

Israel Greenfeld[1*‡], Andrea Camposeo,[2‡] Alberto Portone[2], Luigi Romano[2], Maria Allegrini[2,3], Francesco Fuso[3], Dario Pisignano[2,3*], and H. Daniel Wagner[1]

[1]*Department of Molecular Chemistry and Materials Science, Weizmann Institute of Science, Rehovot 76100, Israel*

[2]*NEST, Istituto Nanoscienze-CNR and Scuola Normale Superiore, Piazza San Silvestro 12, I-56127 Pisa, Italy*

[3]*Dipartimento di Fisica, Università di Pisa, Largo B. Pontecorvo 3, I-56127 Pisa, Italy*

‡ These authors contributed equally

[*] Emails: I.G. green_is@netvision.net.il, D.P. dario.pisignano@unipi.it

S1. Calculation of stretching equation and force function

Stretching equation. In a fast flow, the polymer network is stretched affinely with the flow velocity gradient ∇v. Thus, a segment dL of an entangled chain will elongate at a rate of $dL\nabla v$. In this, we assumed that the flow is uniaxial and sufficiently fast, so that both ∇v and dL are aligned with the direction of the flow. Averaged over the entire primitive length of the chain, L (Figure 1c), the chain will elongate at a rate of $L\nabla v$. This elongation generates an elastic tension in the chain, $F(L) - F(L_0)$, where L_0 is the chain length at equilibrium (at rest). This tension tends to retract the chain at a rate of $-[F(L) - F(L_0)]/\zeta N$, where ζ is the friction constant and N is the number of Kuhn monomers in the chain. Thus, the rate of change in length is

$$\frac{dL}{dt} \approx L\nabla v - \frac{F(L) - F(L_0)}{\zeta N} \tag{S1}$$

The $\approx$ symbol denotes a scaling relationship, where constants of order unity are omitted.

We define the relative chain extension by $\varepsilon = L/L_{\max}$, where $L_{\max} = bN$ is the contour length of a chain having N monomers of (Kuhn) length b. Expressing Equation (S1) in terms of ε, and normalizing the tension force by $k_B T/b$ to make it dimensionless (k_B is Boltzmann constant and T is absolute temperature), we get[1]

$$\frac{d\varepsilon}{dt} \approx \varepsilon \nabla v - \frac{1}{\tau}[f(\varepsilon) - f(\varepsilon_0)] \qquad \text{(S2)}$$

where $\tau \approx \zeta N^2 b^2 / k_B T$ is the relaxation time of a stretched chain in a tube. This is Rouse relaxation time,[2] whose dependence on polymer and solution properties is calculated in section S2. When the electrospinning jet reaches steady state, the velocity gradient is constant, $\nabla v = s$, and the time derivative of the relative extension is zero. Consequently, Equation (S2) reduces to $f(\varepsilon) - f(\varepsilon_0) \approx s\tau\varepsilon$.

An equation similar to Equation (S2), expressed in terms of the stretch ratio $\lambda = L/L_0$, was presented by Bhattacharjee et al. for uniaxial extensional flow,[3] based on the tube model with chain stretching:[4, 5] $d\lambda/dt \approx \lambda\dot{\varepsilon}(S_{11} - S_{22}) - [F(\lambda) - 1]/\tau_R$, where $\dot{\varepsilon}$ is the strain rate (∇v in our terminology), $S_{11} - S_{22}$ is the tube orientation with respect to the flow axis, τ_R is Rouse relaxation time, and $F(\lambda)$ is the tension force normalized by the force at equilibrium. In fast flows such as electrospinning, the tube orientation term tends to unity.[3] Substituting $\lambda = \varepsilon/\varepsilon_0$ (Equation (S3)) and $F(\lambda) = f(\varepsilon)/f(\varepsilon_0)$, and using $f(\varepsilon_0) \cong 3\varepsilon_0$ (Equation (S4)), this equation becomes equivalent to Equation (S2).

Force function. The approximation for the extension dependence of the elastic tension force (dimensionless), $f(\varepsilon) \cong 3\varepsilon(1 - \varepsilon_0)/(1 - \varepsilon)$, is adapted from Ianniruberto and Marrucci.[5] There, the force is defined by $F(\lambda) = [(\lambda_{\max} - 1)/(\lambda_{\max} - \lambda)]\lambda$, where $\lambda = L/L_0$ is the stretch ratio, L is the tube length (primitive path), L_0 is the length at equilibrium, and $\lambda_{\max}$ is the maximum stretch ratio. $F(\lambda)$ is normalized by the equilibrium tension $F(\lambda_0)$. Expressing λ in terms of the relative extension ε:

$$\lambda = \frac{L}{L_0} = \frac{L_{\max}\varepsilon}{L_{\max}\varepsilon_0} = \frac{\varepsilon}{\varepsilon_0} \qquad \text{(S3)}$$

where $\varepsilon = L/L_{\max}$ and $L_{\max}$ is the length of a fully extended chain (contour length). Thus, $\lambda_{\max} = 1/\varepsilon_0$ and $\lambda_0 = 1$. Substituting into the equation of $F(\lambda)$, and normalizing by the force at equilibrium (at rest), $f(\varepsilon_0) \cong 3\varepsilon_0$, we obtain the force function

$$f(\varepsilon) = f(\varepsilon_0)\,F(\varepsilon) = 3\varepsilon_0 \frac{\varepsilon(1-\varepsilon_0)}{\varepsilon_0(1-\varepsilon)} = 3\varepsilon \frac{1-\varepsilon_0}{1-\varepsilon} \quad \text{(S4)}$$

which is the function used in our study. $f(\varepsilon)$ is dimensionless as the force is normalized by $k_B T/b$.

Another known form of the elastic force dependence on extension is the inverse Langevin function, which can be approximated by $f \cong \varepsilon(3-\varepsilon^2)/(1-\varepsilon^2)$.[6] This function has similar properties as the function described in Equation (S4), in that it retrieves the chain linear behavior at small extensions, $f(\varepsilon) \cong 3\varepsilon$, and diverges nonlinearly at high extensions, $f(\varepsilon) \approx (1-\varepsilon)^{-1}$. However, the form in Equation (S4) enables an analytic solution to the stretching equation, and is therefore preferred for use in this study.

S2. Calculation of chain relaxation time and initial extension

Relaxation time. In a semi-dilute solution, a polymer chain consists of successive correlation blobs, which, together with the blobs of neighboring chains, are space-filling without gaps.[7] Consequently, excluded volume interactions of the chain are screened by nearby chains, and it is an ideal chain of blobs. The blob size is $\xi \approx b\varphi^{-\nu/(3\nu-1)}$, where φ is the polymer volume fraction, and ν is Flory's exponent ($1/2$ for a θ-solvent and approximately 0.588 for a good solvent). Each blob contains $g \approx \varphi^{-1/(3\nu-1)}$ Kuhn monomers, and there are N/g blobs in a chain. Because space-filling screens hydrodynamic interactions, the relaxation time can be calculated by the Rouse model:[7]

$$\tau \approx \tau_\xi \left(\frac{N}{g}\right)^2 \quad \text{(S5)}$$

where τ_ξ is the relaxation time of a single blob ξ immersed in a solvent of viscosity η_s. In the length scale of a blob, hydrodynamic interactions are not screened, and therefore τ_ξ is given by the Zimm model:[7]

$$\tau_\xi \approx \frac{\eta_s \xi^3}{k_B T} \tag{S6}$$

The resulting Rouse relaxation time of the full chain is

$$\tau \approx \frac{\eta_s \xi^3}{k_B T}\left(\frac{N}{g}\right)^2 \approx \tau_0 N^2 \varphi^{\frac{2-3\nu}{3\nu-1}} \tag{S7}$$

where $\tau_0 \approx \eta_s b^3/k_B T$ is the relaxation time of a single monomer, and the term $\tau_0 N^2$ is the Rouse chain relaxation time in a dilute solution. The concentration dependence is a power function with an exponent $(2-3\nu)/(3\nu-1)$, which equals 0.31 for a good solvent and 1 for a θ-solvent, the crossover condition between good and poor solvents. Note that, in a semi-dilute entangled polymer network, the relaxation time of a stretched chain scales with the degree of polymerization as N^2, whereas the reptation time for a polymer chain at rest scales as N^3 and is therefore much slower.[7]

Initial extension. The initial extension is given by $\varepsilon_0 \approx L_0/L_{max} \approx l_0/l_{max}$, where $l_0 \approx bN_{e1}^{1/2}\varphi^{-\nu/(3\nu-1)}$ is an entanglement strand end-to-end length for a good solvent and $l_0 \approx bN_{e1}^{1/2}\varphi^{-2/3}$ for a θ-solvent; $l_{max} \approx bN_{e1}\varphi^{-1/(3\nu-1)}$ is a strand's fully extended length for a good solvent and $l_{max} \approx bN_{e1}\varphi^{-4/3}$ for a θ-solvent; N_{e1} is the number of monomers in a strand in a melt, which has a fixed value for a given polymer.[7] Thus, the initial extension of an entangled chain at rest is

$$\varepsilon_0 \approx \frac{l_0}{l_{max}} \approx N_{e1}^{-1/2}\begin{cases}\varphi^{\frac{1-\nu}{3\nu-1}} & \text{good solvent}\\ \varphi^{2/3} & \theta\text{-solvent}\end{cases} \tag{S8}$$

The exponent of φ is 0.54 for a good solvent and 0.67 for a θ-solvent.

S3. Calculation of chain elongation and orientation

In order to calculate the elongation and orientation parameters, we use Equation (7) of the main text to determine the probabilities of monomer directions. Denoting the stretching force in the *y*-direction by $f_y = f(\varepsilon) = f$ and the force at rest by $f_x = f_z = f(\varepsilon_0) = f_0$, one has

$$\begin{aligned} P_y^{\pm} &= e^{\mp f}/2Q \\ P_x^{\pm} = P_z^{\pm} &= e^{\mp f_0}/2Q \end{aligned} \tag{S9}$$

where $Q = \cosh f + 2\cosh f_0$ is a partition function. Substituting for the extension and orientation (Equation (8) in main text)

$$\begin{aligned} &\varepsilon = P_y^+ - P_y^- = -\sinh f / Q \\ &\varepsilon_0 = -1/3 \tanh f_0 \\ &O = 3/2\left(P_y^+ + P_y^-\right) - 1/2 = (\cosh f - \cosh f_0)/Q \end{aligned} \tag{S10}$$

Note that at small initial extension $f_0 \cong 3\varepsilon_0$, and that at high extension f diverges, in agreement with the 3D force equation (Equation (3) in the main text, Equation (S4)). Extracting f and f_0 in terms of ε and ε_0, and substituting into the equation of O, the orientation is obtained in term of the extension[1]

$$O = \frac{-(1 - 3\varepsilon_0^2) + \sqrt{1 - 9\varepsilon_0^2 + 9\varepsilon_0^2\varepsilon^2 + 3\varepsilon^2}}{1 + 3\varepsilon_0^2} \tag{S11}$$

Equation (S11) corresponds to Equation (9) of the main text.

Near a boundary, using Equation (7) of the main text with adorption in the *z*-direction, the probabilities of monomer directions are

$$\begin{aligned} P_y^{\pm} &= e^{\mp f}/2\overline{Q} \\ P_x^{\pm} &= e^{\mp f_0}/2\overline{Q} \\ P_z^{\pm} &= 0 \end{aligned} \tag{S12}$$

where $\overline{Q} = \cosh f + \cosh f_0$ is a partition function. Substituting for the extension and orientation

$$\overline{\varepsilon} = -\sinh f / \overline{Q}$$
$$\overline{\varepsilon}_0 = -1/2 \tanh f_0 \qquad \text{(S13)}$$
$$\overline{O} = (\cosh f - 1/2 \cosh f_0)/\overline{Q}$$

Note that at small initial extension $f_0 \cong 2\overline{\varepsilon}_0$, and that at high extension f diverges, in agreement with the 2D force equation. Extracting f and f_0 in terms of $\overline{\varepsilon}$ and $\overline{\varepsilon}_0$, and substituting into the equation of $\overline{O}$, the orientation is obtained in term of the extension

$$\overline{O} = \frac{-\left(3 - 8\overline{\varepsilon}_0^{\,2}\right) + 3\sqrt{1 - 4\overline{\varepsilon}_0^{\,2} + 4\overline{\varepsilon}_0^{\,2}\overline{\varepsilon}^{\,2}}}{8\overline{\varepsilon}_0^{\,2}} \qquad \text{(S14)}$$

This is Equation (10) of the main text.

The explicit values of f and f_0 are not needed in this modeling, as the resulting orientation functions $O(\varepsilon, \varepsilon_0)$ and $\overline{O}(\overline{\varepsilon}, \overline{\varepsilon}_0)$ are independent of them. Therefore, the orientation analysis applies for a general case of forces, which can account for entropic (elastic) energy due to stretching, as well as for excluded volume repulsive interaction energy to ensure avoidance of multiple lattice occupation. The latter could be incorporated as an approximate mean potential component in the force,[7-9] to represent the potential induced by far monomers and by neighboring chains, without impairing the validity of Equations (S11) and (S14) for a semi-dilute solution.

The orientation Equations (S11) and (S14) do not account for the radial strain rate of magnitude $-s/2$ associated with the longitudinal strain rate s, which exerts radial compression on the polymer chains (see section 3.5 in the main text).[10] We examine now the impact of this compression on the molecular orientation. The compression acts to reduce chain extension in the radial direction from the extension at rest, ε_0, down to a lower value, with a minimal possible extension of ε_0=0 at the jet center. The corresponding minimal radial forces are $f_x = f_z = 0$. The resulting orientation equation at small longitudinal extensions is $O = -1 + \sqrt{1 + 3\varepsilon^2} \cong 3\varepsilon_0^2/2$, a negligible effect ($O$ increases from about 0 to: $O \cong 0.025$ in the MEH-PPV and $O \cong 0.0017$ in the PVP). At high extensions, ε is virtually independent of ε_0 (equation (4) in the main text), and

the effect is negligible as well. A similar argument applies to the orientation of the adsorbed chains, $\overline{O}$.

S4. Scaling approximations for chain elongation and orientation

Approximations for the extension and orientation expressions (Equations (4), (5), (9) and (10) of the main text) are useful, as they provide simple scaling relationships with respect to the relative strain rate, s/s_c. The following approximations were obtained by series expansions at small ε_0. The chain extension scales as

$$\begin{aligned} &\varepsilon \cong \varepsilon_0\left(1+\frac{s}{s_c}\right) && s \ll s_c \\ &\varepsilon \cong 1-\left(\frac{s}{s_c}\right)^{-1} && s \gg s_c \\ &\varepsilon = \varepsilon_0^{1/2} && s = s_c \end{aligned} \qquad \text{(S15)}$$

where s_c is $3/\tau$ far from a boundary and $2/\tau$ near a boundary, and $\overline{\varepsilon}_0$ is used near a boundary. Far from the boundary of a filler, the orientation scales as

$$\begin{aligned} &O \cong 3\varepsilon_0^2\frac{s}{s_c} && s \ll s_c \\ &O \cong 1-\frac{3}{2}\left(\frac{s}{s_c}\right)^{-1} && s \gg s_c \\ &O \cong \frac{3}{2}\varepsilon_0 && s = s_c \end{aligned} \qquad \text{(S16)}$$

where $s_c = 3/\tau$. Near the boundary of a filler, the orientation scales as

$$\begin{aligned} &\overline{O} \cong \frac{1}{4}+\frac{3}{2}\overline{\varepsilon}_0^2\frac{s}{\overline{s}_c} && s \ll \overline{s}_c \\ &\overline{O} \cong 1-\frac{3}{2}\left(\frac{s}{\overline{s}_c}\right)^{-1} && s \gg \overline{s}_c \\ &\overline{O} \cong \frac{1}{4}+\frac{3}{4}\overline{\varepsilon}_0 && s = \overline{s}_c \end{aligned} \qquad \text{(S17)}$$

where $\overline{s}_c = 2/\tau$. Note that at the critical strain rate the orientation depends only on the initial extension.

S5. Additional experimental data of molecular and optical anisotropy

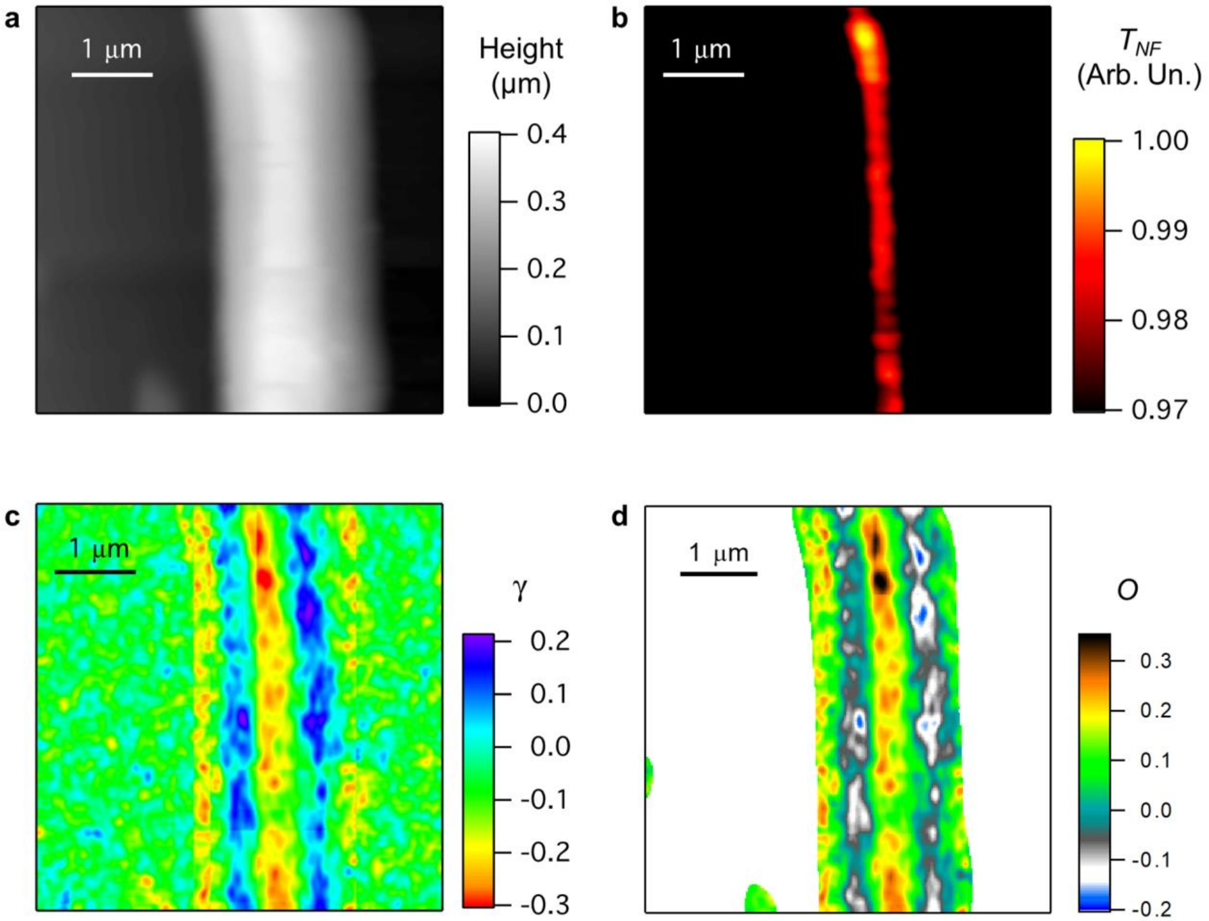

Figure S1. **a** Topography map of a single PVP/MEH-PPV/WO_3 electrospun fiber. **b-c** Corresponding maps of the near field excited optical extinction signal by the nanofiber, (T_{NF}) (b), and of the dichroic ratio, γ (c). **d** Map of Herman's orientation parameter O derived from the data shown in (c) and following the methodology described previously:[11] $O = \frac{R-1}{R+2}\frac{2}{3\cos^2(\alpha)-1}$, where $R = \frac{I_{\parallel}}{I_{\perp}} = \frac{1-\gamma}{1+\gamma}$ and α is the angle formed by the direction of the fiber length and a reference direction that is experimentally determined (α is about 25 degrees).

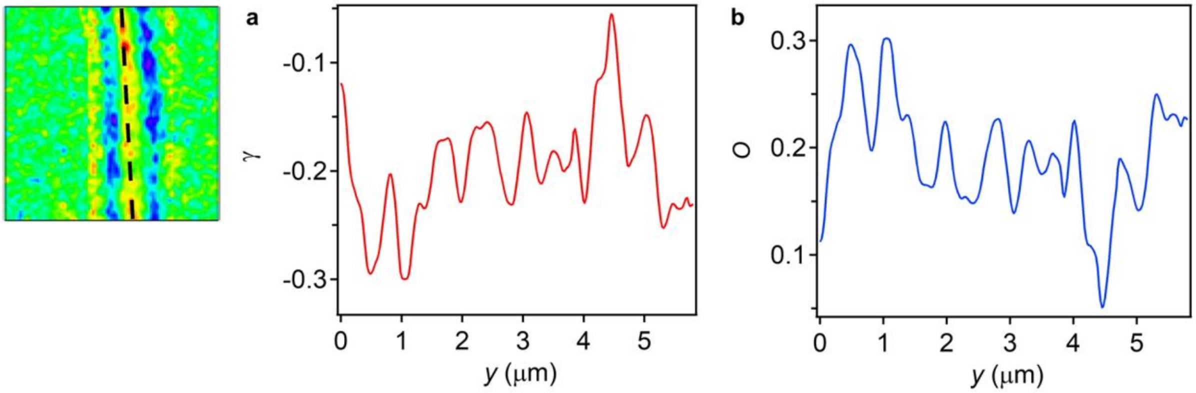

Figure S2. Line profile analysis of γ (**a**) and of the Herman parameter O (**b**) displaying the cross section along the vertical direction, y (positive downward), which is highlighted as dashed segment in the inset image shown on the left.

References

1. Greenfeld, I.; Sui, X. M.; Wagner, H. D., Stiffness, Strength, and Toughness of Electrospun Nanofibers: Effect of Flow-Induced Molecular Orientation. *Macromolecules* **2016,** *49* (17), 6518-6530.

2. Doi, M.; Edwards, S. F., *The theory of polymer dynamics*. the Clarendon Press, Oxford University Press, New York, : 1986.

3. Bhattacharjee, P. K.; Nguyen, D. A.; McKinley, G. H.; Sridhar, T., Extensional stress growth and stress relaxation in entangled polymer solutions. *J Rheol* **2003,** *47* (1), 269-290.

4. Pearson, D. S.; Kiss, A. D.; Fetters, L. J., Flow-Induced Birefringence of Concentrated Polyisoprene Solutions. *J Rheol* **1989,** *33* (3), 517-535.

5. Ianniruberto, G.; Marrucci, G., A simple constitutive equation for entangled polymers with chain stretch. *J Rheol* **2001,** *45* (6), 1305-1318.

6. Cohen, A., A Pade Approximant to the Inverse Langevin Function. *Rheol Acta* **1991,** *30* (3), 270-273.

7. Rubinstein, M.; Colby, R. H., *Polymer physics*. Oxford University Press: Oxford ; New York, 2003; p xi, 440 p.

8. Flory, P. J., *Principles of Polymer Chemistry*. Cornell University Press: Ithaca, New York, 1953.

9. de Gennes, P. G., *Scaling concepts in polymer physics*. Cornell University Press: Ithaca, N.Y., 1979; p 324 p.

10. McKinley, G. H.; Sridhar, T., Filament-stretching rheometry of complex fluids. *Annu Rev Fluid Mech* **2002,** *34*, 375-415.

11. Papkov, D.; Delpouve, N.; Delbreilh, L.; Araujo, S.; Stockdale, T.; Mamedov, S.; Maleckis, K.; Zou, Y.; Andalib, M. N.; Dargent, E.; Dravid, V. P.; Holt, M. V.; Pellerin, C.; Dzenis, Y. A., Quantifying Polymer Chain Orientation in Strong and Tough Nanofibers with Low Crystallinity: Toward Next Generation Nanostructured Superfibers. *Acs Nano* **2019,** *13* (5), 4893-4927.
